# The association of the Affordable Care Act with nutrient consumption in adults in the United States

**DOI:** 10.3389/fpubh.2023.1244042

**Published:** 2023-12-22

**Authors:** Hilary Kirk, Theresa A. Tufuor, Amy L. Shaver, Jing Nie, Prasad P. Devarshi, Keri Marshall, Susan Hazels Mitmesser, Katia Noyes

**Affiliations:** ^1^Division of Health Services Policy and Practice, Department of Epidemiology and Environmental Health, School of Public Health and Health Professions, University at Buffalo, Buffalo, NY, United States; ^2^Department of Pharmacy Practice, School of Pharmacy and Pharmaceutical Sciences, University at Buffalo, Buffalo, NY, United States; ^3^Pharmavite LLC, West Hills, CA, United States

**Keywords:** nutritional supplements, Affordable Care Act, cancer, nutritional status, National Health and Nutrition Examination Survey

## Abstract

The Patient Protection and Affordable Care Act, more commonly known as the ACA, was legislation passed in the United States in 2010 to expand access to health insurance coverage for millions of Americans with a key emphasis on preventive care. Nutrition plays a critical role in overall wellness, disease prevention and resilience to chronic illness but prior to the ACA many Americans did not have adequate health insurance coverage to ensure proper nutrition. With passage of the ACA, more individuals received access to nutritional counseling through their primary care physicians as well as prescription vitamins and supplements free of charge. The objective of this study was to evaluate the impact of a national health insurance reform on nutrient intake among general population, including more vulnerable low-income individuals and patients with chronic conditions. Using data from the National Health and Nutrition Examination Survey (NHANES), we identified 8,443 adults aged 21 years and older who participated in the survey before (2011–2012) and after the ACA (2015–2016) implementation and conducted a subgroup analysis of 952 respondents who identified as Medicaid beneficiaries and 719 patients with a history of cancer. Using pre-post study design and bivariate and multivariable logistic analyses, we compared nutrient intake from food and supplementation before and after the ACA and identified risk factors for inadequate intake. Our results suggest that intake of micronutrients found in nutrient-dense foods, mainly fruit and vegetables, has not changed significantly after the ACA. However, overall use of nutritional supplements increased after the ACA (*p* = 0.05), particularly magnesium (OR = 1.02), potassium (OR = 0.76), vitamin D (both D2, and D3, OR = 1.34), vitamin K (OR = 1.15) and zinc (OR = 0.83), for the general population as well as those in our subgroup analysis Cancer Survivors and Medicaid Recipients. Given the association of increased use of nutritional supplements and expansion of insurance access, particularly in our subgroup analysis, more research is necessary to understand the effect of increasing access to nutritional supplements on the overall intake of micro- and macronutrients to meet daily nutritional recommended allowances.

## 1 Introduction

The World Health Organization, in collaboration with United Nation’s Children’s Fund (UNICEF), Food and Agricultural Organization of the United Nations (FAO) and World Food Program (WFP) has emphasized the role of public policy in modifying food systems to improve people’s nutrition and health (1). Although global food production of calories has kept pace with population growth, the common prioritization of quantity and profitability over nutritional value has meant healthy diets remain unaffordable for over 40% of the world’s population. At the same time, a surplus of availability of highly processed foods, which are often calorie-dense but nutrient-poor, contribute to the alarming rise in diet-related diseases, such as diabetes, heart disease and certain cancers. The Patient Protection and Affordable Care Act, a public policy more commonly known as the ACA, was passed in the United States in 2010 to expand access to health insurance coverage for millions of Americans. Historically the United States spends far more on health care than comparable countries, but with less optimal health outcomes. This is partly due to years of underinvestment in preventive health services (2). The ACA also expanded access through the authorization of Medicaid expansion in many states to address issues of health equity in vulnerable communities. Medicaid is a public health insurance program jointly funded by federal and state governments to provide coverage for low-income Americans (3). The ACA is widely considered one of most significant regulatory overhauls of the United States health care industry by requiring insurance companies to reprioritize preventive health services through free-of-charge coverage for what are deemed “Essential Health Benefits.” These mandated services include the provision of wellness and preventive care, including blood pressure screenings, cholesterol checks, cancer screenings, vaccinations, and nutrition counseling for all individuals (3, 4).

Prior to the passage of the ACA, previous studies of nutrient intake using national survey data suggested that the majority of the US adult population did not meet the recommended daily intake of nutrient-dense foods which may contribute to high rates of chronic diseases, such as hypertension and type 2 diabetes (5). Starting in 2016 the ACA required most health plans, including Medicaid programs, to cover annual nutrition counseling for people at high risk of chronic diseases at no charge, which previouly were not universally covered (6). The ACA further expanded the Prevention and Public Health Fund which provided direct funding in communities to provide chronic disease self-management and diabetes prevention programming, as well as funding for preventing health services programming (7). Many of these initiatives included access to nutritional counseling provided by registered dietitians and other trained practitioners.

In addition to being a vital component of general health and wellness and staying healthy, nutrition therapy can be an important component of adjunct care in treating most all disease states including obesity, heart failure, diabetes, hypertension, COPD and arthritis (8–10). Specifically, cancer patients are at significant risk for malnutrition and cachexia because cancer and cancer treatments have a serious negative impact on a patient’s ability to consume and absorb food and nutrients (11, 12). Cancer patients are at risk throughout the, treatment continuum and into survivorship often falling prey to nutrient shortfalls (11). Therefore, dietary supplements have been used to treat cancer patients and patients with cardiovascular disease. Dietary supplements have been found to reduce the risk of cardiovascular disease, cancer, and all-cause mortality (13). A recent study by Shaver and colleagues found that patients with cancer that use nutrition supplements are less likely to be hospitalized and have a lower probability of dying post follow-up (14). More specifically, calcium supplementation has been found to reduce the risk of cancer in some patients, particularly lung cancer patients (15, 16). In 2017 the American Society for Nutrition encouraged more research on preventive health service policies particularly that examine the role nutrition and nutritional supplementation play in disease prevention and chronic care management (17).

To our knowledge, this is the first study that examined the association with the Affordable Care Act on nutrient intake among adult Americans. We hypothesize that after the ACA, more patients received nutritional counseling that in turn, led to better food choices and/or increased supplement intake to improve overall health and wellbeing. Using data from the National Health and Nutrition Examination Survey (NHANES), an ongoing national initiative, our study assessed whether the improved access to preventive care under a national health insurance reform (the ACA), improved the overall intake of the key nutrients among general population and low income individuals and patients with chronic nutrition-related diseases. In our analysis, we examined nutrients consumed with food separately from nutritional supplementation to account for differences in demand and consumption behavior regarding general goods and services, like food, and insurance-subsidized services like prescription nutrition supplementation.

## 2 Methods

### 2.1 Study design and population

The study was based on the data from the National Health and Nutrition Examination Survey (NHANES). The National Health and Nutrition Examination Survey (NHANES) is designed to assess the health and nutritional status of adults and children in the United States. The survey is unique in that it combines interviews and physical examinations. NHANES is a major program of the National Center for Health Statistics (NCHS) that receives millions of dollars from the federal budget. NCHS is part of the Centers for Disease Control and Prevention (CDC) and has the responsibility for producing vital and health statistics for the Nation. Several hundreds of highly trained home interviewers collect and encrypt data on laptops, use printed materials to prompt and verify responses, and verify prescription medicine use by examining container labels. The NHANES is a survey conducted annually by the National Center for Health Statistics (NCHS) housed within the Centers for Disease Control and Prevention (CDC) since 1960s. According to the CDC, the survey is a collection of physical examinations and interviews with a nationally representative sample of 5000 United States citizens. It is used to monitor the health and nutritional status of non-institutionalized individuals in the US (18). NHANES uses a representative sample of noninstitutionalized U.S. civilians, selected by a complex, multistage probability design. Briefly, participants were interviewed in their homes and subsequently examined in mobile examination centers (MEC) in 15 U.S. geographic locations. All participants provided informed consent prior to data collection. The survey uses stratified, multistage probability sampling to obtain nationally representative samples for each year; data from questionnaire and laboratory tests are released every 2 years (18).

Adults aged 21 years and older with non-missing values on nutrient intake from diet (which includes both food and drink) and supplements were included in our study. The final sample included 8,443 participants that corresponded to a weighted sample of 223,400,729 individuals for the years 2011–2012 and 232,006,739 for the years 2015–2016. The study is in compliance with the University at Buffalo Institutional Review Board policies. The subgroup analysis examined 952 patients who reported receiving Medicaid benefits, with 444 patients in 2011–12 and 508 in 2015–2016.

### 2.2 Nutrient intake measures

We hypothesize that after the ACA, more patients received nutritional counseling that in turn, led to better food choices and/or increased supplement intake to improve overall health and wellbeing. Nutrient intake from food was estimated based on two dietary recalls. All NHANES participants are eligible to participate in two 24-h dietary recall interviews performed first in the Mobile Examination Center and second telephonically within 3–10 days of the medical examination (19, 20). Nutrient intake from supplements was ascertained in the participant’s home before physical examination. Dietary supplement information is collected by trained interviewers. An affirmative response to supplement use is followed by an examination of the supplement containers by the interviewer who enters the product name and strength into the Computer-Assisted Personal Interviewing (CAPI) system. This information is later reviewed by trained nutritionists at the NCHS to discern the exact product reported by participants and calculate daily average intake of nutrients based on the previous 30  days’ food, supplement, and antacid intake.

Sufficient nutritional intake was determined based on the US recommended daily allowances (RDA) and other standards (19–23). Information was gathered on the following nutrients: calcium, folate (as dietary folate equivalent or, DFE), dietary fiber, lycopene, magnesium, potassium, selenium, lutein + zeaxanthin, vitamin B6, vitamin B12, vitamin C, vitamin D (D2 and D3), vitamin K, and zinc. These nutrients were chosen due to previous research indicating their utility in cancer prevention and immune health (23–36). RDA was chosen so as to make the study more translatable to the general public as well as to be translatable to the labeling as found on many supplements.

### 2.3 Participant characteristics

Nutritional intake can vary by individual personal characteristics and socio-economic status (5, 37–40). Demographic details of participants, such as age, gender, and race were provided in an interview. Social characteristics were also provided via interview and included: smoking status categorized as current, former, and never smoker; level of education categorized as ≤high school graduate, some college, and ≥ Bachelor’s degree. Participants answered whether they were US citizens or not. Socioeconomic status (SES) was assessed in terms of income to poverty ratio (IPR), and was categorized as <100%, 100 to <200%, 200 to <300%, and ≥ 300%. Health insurance was categorized as Private, Medicare, Medicaid, Other and None. Body mass index (BMI) was measured as part of the anthropometric examination and was utilized to calculate obesity, defined as a BMI of greater than or equal to 30. The medical conditions questionnaire is asked in the home by a trained interviewer utilizing the CAPI system with questions stratified by gender (for conditions related to specific anatomy) and age. Participants were asked if they had ever been told they had cancer, arthritis, diabetes, congestive heart failure (CHF), chronic obstructive pulmonary disease (COPD), or hypertension.

### 2.4 Statistical analysis

All analyses were conducted using appropriate survey weights in SAS 9.4. Descriptive analyses of continuous variables included calculating the means (SD) and frequencies (%) of anthropometric, medical, and categorical sociodemographic characteristics of the participants. Propensity score (PS) matching was utilized to assist in controlling for any differences in the survey sample between 2011–2012 and 2015–2016 cohorts. Participants from each time period were matched on age, gender, race/ethnicity, level of education, income, comorbidities, and citizenship status. After PS matching, there were no statistically significant differences in the sample by the key covariates; the final sample for analysis included 7309 patients.

We used binary logistic regressions stratified and clustered by the NHANES survey strata (PSU). The ACA variable is a binary variable based on the NHANES cohort, since the ACA is a national policy. With respect to nutritional intake, the outcome variable was defined as whether or not an individual consumed a recommended daily amount (RDA), for each nutrient. We incorporated survey weights to extrapolate the survey results from the subsample of the NHANES participants who completed a 2-day dietary recall (SAS proc surveylogistic) to the national community population. Odds ratios (Post- vs. Pre-ACA) were adjusted for the participants age, sex, race, education, income to poverty ratio, comorbidities (arthritis, diabetes, CHF, COPD, HTN, obesity cancer), US citizenship, and smoker status to control for the independent association with these factors on nutritional intake (5, 37–40). Total nutrient intake combined the intake of both food and supplement. A regression analysis was run for each type of nutrient intake against total nutrient intake at significance *p* < 0.05.

We also evaluated nutrient consumption of patients with nutrition-related comorbidities that increased in prevalence during the time of the study (arthritis, diabetes, obesity and cancer, Table 1). Because cancer patients are at significant risk for malnutrition analyses were conducted on a subgroup of 719 cancer survivors from the matched sample. The cancer survivors were individuals who reported being told they had cancer. Similar analyses were conducted on the subgroup of 952 Medicaid beneficiaries from the matched sample, because of the significant regulatory overhaul of the program due to the ACA. Descriptive statistics, mean and standard error were calculated for a sample of Medicaid patients included in the NHANES population surveyed. A test of differences was run examining survey respondents reported nutrient intake from dietary recall and nutrient intake from supplements.

**Table 1:** Population characteristics before and after the ACA (*n* = 8,443), weighted results, by percent.

Characteristic	Pre-ACA (2011–2012) n = 223,400,729	Post-ACA (2015–2016) *n* = 232,006,739	value of *p*
Age	47.4 (0.9)	48.3 (0.7)	0.46
Sex			0.77
Male	48.2	48.6	
Female	51.8	51.4	
Race			0.93
Mexican American	8.2	8.7	
Other Hispanic	6.0	5.8	
NH White	67.0	65.3	
NH Black	11.4	10.7	
Other	7.3	9.5	
Education			0.82
≤High School Graduate	36.3	34.3	
Some College	31.7	33.5	
≥Bachelor’s Degree	32.1	32.1	
Income to Poverty Ratio			0.06
<100% FPL	17.9	13.6	
100 to <200% FPL	20.7	19.0	
200 to <300% FPL	14.1	16.8	
> = 300% FPL	38.3	38.6	
Refused to answer	8.9	12.0	
Comorbidities			
Arthritis	23.6	28.3	0.04
Diabetes	9.3	11.5	0.03
CHF	2.6	2.4	0.58
COPD	7.5	3.3	<0.001
HTN	31.8	32.2	0.85
Obesity	34.6	39.8	0.03
Cancer	9.9	12.4	0.02
US Citizen	82.9	82.8	0.98
Regular Healthcare Provider	85.2	83.5	0.35
Smoker			0.37
Current	20.0	17.6	
Former	24.2	25.9	
Never	55.9	56.4	
Insurance			0.02
Private	60.9	61.0	
Medicare	7.5	9.0	
Medicaid	6.8	8.6	
Other	6.0	8.2	
None	18.7	13.2	
Dietary Supplements			0.05
No	46.8	41.5	
Yes	53.2	58.5	

CHF, congestive heart failure; COPD, chronic obstructive pulmonary disease; HTN, hypertension; FPL, federal poverty limit; NH, non-Hispanic.

Data presented as frequency (%) or mean (SD); p represents results of *t*-test or chi-square test of difference as applicable.

## 3 Results

### 3.1 Characteristics of the study cohort

The mean age was similar to the general population (45.7 pre and 43.8 years post ACA, *p* = 0.32). The proportion of Medicaid recipients increased from 6.8% of the respondents prior to the ACA to 8.6% in the post-ACA cohort (*p* = 0.02). After the ACA, fewer individuals reported not having insurance coverage (18.7% vs. 13.2%, *p* = 0.02) mainly due to the increase in Medicaid expansion. Patient demographics did not change significantly over the study time period (Table 1). The study participants were on average 47.4 years old prior to the ACA and 48.3 years old after the ACA, predominantly Non-Hispanic White (67.0% vs. 65.3%), female (51.8 and 51.4%), and privately insured (60.9% vs. 61.0%). Most participants reported having a regular source of primary care (85.2% vs. 83.5%; *p* = 0.35).

The proportion of participants reporting using at least one dietary supplement increased from 53.2 to 58.5%, *p* = 0.05. Prevalence of several chronic diseases related to nutritional status also increased during this time (arthritis, diabetes, obesity, and cancer, *p* < 0.05), while prevalence of COPD decreased (7.5% vs. 3.3%, *p* < 0.001). After propensity score matching, there were no significant differences in the comorbidities and smoking status of the participants before and after the ACA.

### 3.2 Evaluation of nutrient intake

Table 2 presents the average total daily nutrient intake as percent of RDA by ACA status by intake source (food vs. dietary supplements). For several nutrients, the average intake exceeded the RDA before the ACA and remained high afterwards. For instance, the intake of vitamin D was above RDA before the ACA (103% RDA) and further increased to 141% RDA (*p* = 0.01) post-ACA as vitamin D supplementation increased from 70% pre-ACA to 77% post-ACA. Other nutrients with increased intake included calcium, folate, selenium, vitamins B6, B12, C, and K, and zinc, and were mainly obtained from food.

**Table 2:** Average daily nutrient intake from food and supplements by ACA status.

Nutrients	RDA	Pre-ACA			Post-ACA			*p^^^*
		Average DNI (mean, SE)	% of RDA	% from supplements	Average DNI (mean, SE)	% of RDA	% from supplements	
Vitamin D (D2 + D3) (mcg)	15–20 μg/d	15.4 (1.0)	103	70	20.7 (1.7)	141	77	0.01
Potassium (mg)	2600–3400 μg/d	2778.8 (39.5)	93	<1	2617.9 (46.4)	87	<1	0.01
Calcium (mg)	1000–1300 μg/d	1134.6 (22.7)	114	15	1077.9 (27.3)	109	13	0.12
Folate, DFE (mcg)	400 μg/d	784.3 (15.6)	196	28	741.4 (28.0)	187	30	0.19
Dietary fiber (gm)	21–38 g/d	18.3 (0.4)	58	<1	17.3 (0.5)	55	<1	0.11
Lycopene (mcg)	10,000	5485.7 (246.8)	55	2	5325.8 (256.1)	53	1	0.66
Magnesium (mg)	320–420 μg/d	339.1 (6.6)	92	9	337.9 (10.5)	91	10	0.92
Selenium (mcg)	55 μg/d	146.8 (16.1)	267	22	127.9 (2.3)	232	11	0.25
Lutein + zeaxanthin (mcg)	12,000	2093.8 (220.4)	17	18	1947.4 (131.8)	16	16	0.57
Vitamin B6 (mg)	1.3–1.7 mg/d	5.8 (0.4)	442	62	4.9 (0.6)	368	57	0.20
Vitamin B12 (mcg)	2.4 μg/d	67.3 (10.6)	2804	91	85.2 (10.8)	3675	93	0.25
Vitamin C (mg)	75–90 mg	175.2 (8.8)	212	51	169.5 (10.8)	208	55	0.69
Vitamin K (mcg)	90–120 μg/d	133.9 (9.2)	128	5	128.3 (4.9)	122	6	0.60
Zinc (mg)	8–11 μg/d	15.6 (0.3)	164	27	15.3 (0.4)	162	27	0.51

*p*^ indicates statistically significant change in nutrient intake of all NHANES participants pre and post ACA implementation.

At the same time, intake of many core nutrients remained under the recommended amount. Many participants with nutrient intake below RDA from food, also did not use enough supplementation to fill the nutrient gap from food by reaching the nutrient-specific RDA goal neither pre- nor post-ACA. Potassium intake declined from 93% RDA pre-ACA to 87% RDA post-ACA (*p* = 0.01) and came primarily from food. Intake of dietary fiber, lycopene and lutein and zeaxanthin remained below 60% RDA before and after the ACA, with minimal supplementation.

### 3.3 Subgroup analysis

We conducted a multivariable adjusted regression analysis for a subgroup of Medicaid beneficiaries, a population subgroup that grew the most under the ACA (Table 3). Medicaid beneficiaries reported significantly more nutritional deficiencies than the general population (for calcium, vitamin D and zinc). Similar to the general population, there was an increase in the number of people reporting dietary supplement intake after the ACA. Nutritional intake of micro and macro-nutrients among Medicaid beneficiaries significantly increased for five of the 15 core nutrients, mainly due to an increase in the use of nutritional supplements (Magnesium, Potassium, Vitamin D (D2 + D3), Vitamin K, and Zinc). Most notably intake of Vitamin D (D2 + D3) showed a statistically significant increase for overall increase in nutritional intake and increase in intake from supplementation, where respondents reported a 47.9% increase in Vitamin D supplement intake from 2011 to 2011 (16.03 μg/d) to 2015–2016 (30.77 μg/d) (*p* < 0.05). Similarly, the increase in total Magnesium intake pre- versus post-ACA, was primarily due to statistically significant increase in magnesium supplementation (66.98, 142.33, *p* = <0.05, respectively). Despite the increases, the total intake of Vitamin D and Magnesium still remained below current RDA recommendations at 15–20 μg/d and 320–420 μg/d, respectively. Considerably, intake for Vitamin K and Zinc increased post-ACA and levels were within RDA limits.

**Table 3:** Medicaid status nutrient intake^.

		2011–2012			2015–2016				
	RDA	Total mean	Food mean	Suppl mean	% suppl	Total mean	Food mean	Suppl mean	% suppl
Calcium (n)	1000–1300 μg/d	989.25 (444)	897.72 (444)	302.96 (131)	30.21%	958.06 (508)	854.30 (508)	350.60 (146)	29.60%
Folate (n)	400 μg/d	623.42 (444)	496.38 (444)	591.41 (95)	21.40%	662.20 (508)	455.70 (508)	880.21 (113)	22.24%
Fiber (n)	21–38 g/d	15.09 (444)	15.08 (444)	0.46 (2)	0.35%	13.97 (508)	13.93 (508)	3.32 (6)	1.16%
Lycopene (n)		4804.55 (386)	4809.62 (382)	711.63 (18)	4.66%	4305.22 (444)	4290.50 (440)	504.23 (39)	8.78%
Magnesium (n)	320–420 μg/d	274.49 (444)	262.37 (444)	66.98 (83)	18.69%	277.18 (508)	249.02 (508)	142.33* (88)	17.32%
Potassium (n)	2600–3400 μg/d	2397.37 (444)	2391.90 (444)	50.42 (52)	11.71%	2245.63 (508)	2236.73 (508)	92.81* (58)	11.42%
Polyunsaturated Fatty Acids (n)	6–8 g/d	16.75 (444)	16.74 (444)	0.80 (8)	1.80%	16.92 (508)	16.90 (508)	0.68 (17)	3.35%
Selenium (n)	55 μg/d	106.69 (444)	100.74 (444)	40.86 (67)	15.09%	111.85 (508)	103.63 (508)	54.66 (80)	15.75%
Lutein + Zeaxanthin (n)		1230.09 (444)	1188.75 (444)	883.07 (21)	4.73%	1435.03 (508)	1383.02 (508)	708.72 (42)	8.27%
Vitamin B6 (n)	1.3–1.7 mg/d	4.32 (444)	1.94 (444)	11.19 (94)	21.17%	3.16 (508)	1.83 (508)	5.97 (110)	21.65%
Vitamin B12 (n)	2.4 μg/d	43.43 (444)	6.10 (444)	166.35 (101)	22.75%	40.01 (508)	5.42 (507)	135.12 (125)	24.61%
Vitamin C (n)	75–90 mg	136.08 (444)	80.37 (444)	227.69 (108)	24.32%	145.68 (508)	74.5 (508)	273.19 (121)	23.82%
Vitamin D (D2 + D3) (n)	15–20 μg/d	9.84 (443)	4.77 (442)	16.03 (127)	31.44%	14.11 (505)	4.71 (504)	30.77* (146)	30.44%
Vitamin K (n)	90–120 μg/d	92.63 (444)	89.22 (444)	25.24 (64)	14.41%	109.33 (508)	102.28 (508)	48.16* (73)	14.37%
Zinc (n)	90–120 μg/d	12.19 (444)	10.06 (444)	10.48 (86)	19.37%	12.76 (508)	9.59 (508)	14.92* (107)	21.06%

^Comparing nutrient intake from food consumption and use of supplements between 2011–2012 and 2015–2016.

*Statistically significant results for change in mean amount of supplementation *α* = 0.05.

No significant changes pre-post ACA in nutrient intake and supplementation were detected in subgroups of patients with these disorders except for cancer (Table 4). After the ACA, beneficiaries with cancer history were significantly more likely to take adequate (RDA) amount of lycopene, but less likely to consume enough folate or vitamin B12. While the odds of consuming sufficient dosage of vitamin D did not change among patients with cancer after the ACA passage, the general population was more likely to have an intake of vitamin D at or above RDA recommendations (OR: 1.34; 95% CI [1.10–1.63]; *p* = 0.005).

**Table 4:** ACA status and odds of reaching RDA nutrient intake.

	General population (*n* = 7,308)			Cancer survivors (*n* = 719)		
Nutrient	Adjusted odds ratio*	95% CI	*p*	Adjusted odds ratio*	95% CI	*p*
Calcium	0.88	(0.73–1.06)	0.16	1.06	(0.65–1.73)	0.82
Folate, DFE	0.74	(0.60–0.91)	0.01	0.46	(0.30–0.73)	0.001
Dietary fiber	0.94	(0.75–1.19)	0.60	1.01	(0.54–1.90)	0.97
Lycopene	1.08	(0.88–1.34)	0.44	2.21	(1.09–4.49)	0.03
Magnesium	1.02	(0.83–1.26)	0.84	1.32	(0.79–2.22)	0.28
Potassium	0.76	(0.63–0.91)	0.005	1.07	(0.65–1.75)	0.79
Selenium	0.98	(0.75–1.27)	0.85	1.07	(0.58–2.00)	0.82
Lutein + zeaxanthin	0.78	(0.47–1.29)	0.32	0.82	(0.21–3.22)	0.77
Vitamin B6	0.86	(0.70–1.06)	0.16	0.96	(0.59–1.58)	0.88
Vitamin B12	0.77	(0.64–0.92)	0.01	0.54	(0.32–0.90)	0.02
Vitamin C	1.02	(0.82–1.26)	0.86	1.42	(0.85–2.36)	0.17
Vitamin D (D2 + D3)	1.34	(1.10–1.63)	0.005	1.69	(0.99–2.86)	0.052
Vitamin K	1.15	(0.94–1.40)	0.17	1.25	(0.73–2.14)	0.42
Zinc	0.83	(0.70–0.99)	0.04	0.75	(0.47–1.18)	0.21

^*^ORs (Post- vs Pre-ACA) adjusted for age, sex, race, education, income to poverty ratio, comorbidities (arthritis, diabetes, CHF, COPD, HTN, obesity cancer), US citizen, and smoker status. Total nutrient intake combines the intake of both food and supplement.

## 4 Discussion

In this population-based study, we examined changes in individual nutritional intake that were enabled by the national health insurance reform as a part of the 2010 ACA. We demonstrated a significant increase in the use of some dietary supplements among the general population as well as for low-income populations post-ACA era, especially for vitamin D. Notwithstanding, many Americans continue to not meet the RDA for potassium, dietary fiber, and magnesium, despite improvements in insurance coverage and increased availability of no-cost nutrition counseling. Medicaid beneficiaries reported significantly more nutrient deficiencies than the general population (for calcium, vitamin D and zinc) but also demonstrated a greater increase in use of dietary supplements post-ACA (for calcium, folate, fiber, magnesium, potassium, vitamins D and K and zinc). Patients with a history of cancer demonstrated better intake of lycopene and lutein, nutrients known to have protective effects in cancer, but were less likely to consume enough of other core nutrients (folate and B12) (41).

Several studies have suggested a positive impact of the health insurance expansion on the use of preventive services among the highest risk populations, mainly due to the affordability of these services. Smoking cessation uptake was shown to be higher following the ACA than prior to its inception, and those enrolled in Medicaid were more likely to quit smoking than those with commercial insurance (OR 1.49; 95% CI 1.29, 1.73) (37). Moreover, despite current economic theory that suggests new access to health insurance can create *ex ante* moral hazard, Cotti and colleagues found that low-income individuals who benefitted from the public insurance expansion due to the ACA actually engaged in healthier health habits (38). A population-based observational study assessed the impact of the ACA on food insecurity and found that Medicaid expansion following ACA implementation was associated with a 2.2 percentage-point decline in very low food security (VLFS) (39). This study concluded that low-income families experienced less food insecurity because out-of-pocket health care expenses were reduced because of Medicaid expansion, thereby increasing expendable income to purchase food.

One possible explanation for the limited impact of the ACA on the general population nutrition status is a lack of patient knowledge surrounding benefits of coverage of available nutrition services (40). Additionally, primary care providers do find value in nutrition counseling but often feel poorly trained to discuss nutrition with their patients, and too short on time to engage with their patients (42, 43). Individuals obtained most of their nutrients from food, with the exception of vitamins C, D, B6 and B12 which came mostly from supplementation which is similar to a recent finding by Devarshi et al. (44). Patients with history of cancer are more likely to receive nutrition counseling than the general population as a part of their multidisciplinary cancer and survivorship care but may be more inclined to focus on cancer survival than general wellness (45, 46).

One potential explanation for the increased intake in vitamin D post-ACA is the increase in the number of reports of its association with various disease states and conditions that accidently happened during the same time as the ACA reform. Vitamin D has been studied and shown to prevent falls in older adult patients, reduce fracture risk in post-menopausal women and in individuals with osteoporosis (47–50). Similarly, the increased proportion of cancer patients with adequate lycopene intake may be reflective of its publicized role as an antioxidant for cancer, specifically prostate cancer (51, 52). With research studies and social media reports supporting the use of vitamin D and lycopene, clinicians may be more likely to recommend their intake in specific patient populations, as a result of having a more informed understanding of their benefits. In addition to clinicians, others involved in wellness programs, such as insurance companies and employers, may also play a role in educating their clients and employees about the benefits of adequate nutrition.

The reduction in potassium intake post-ACA, and the corresponding reduction in the number of individuals with sufficient intake of potassium, vitamin B12 and folate may be related to the reduction in consumption of foods enriched with these nutrients. According to the US National Institutes of Health, top sources of potassium among American adults are milk, potatoes, coffee, tea and other nonalcoholic beverages, but potassium can also be found in fruits and vegetables such as bananas. Folate is commonly found in fortified cereals, grains, bread and pasta, and well as dark green veggies including asparagus. Shan et al. studied food trends among American adults from the years 1999 to 2016, and found a decrease in consumption of low-quality carbohydrates, which included potatoes and beverages with added sugars (53). In studying trends in food intake, many higher income countries fall short on adequate fruit and vegetable intake (54). In the wake of increasing awareness of healthy eating, Americans may be curbing the intake of foods known as low-quality carbohydrates (such as potatoes, breads, and pasta) which are significant sources of potassium and folate, without adequately adding alternative sources, such as fruits and vegetables.

Data from the National Health and Nutrition Examination Survey, 2015–2018 reports that the percentage of adults who consumed any fruit on a given day has decreased over time, from 77.2% in 1999–2000 to 64.9% in 2017–2018 (55). Moreover, statistics indicate that the ability to access fresh produce is directly associated with income levels. In fact, NHANES continues to report that “the percentage of adults who consume any vegetables increased with increasing family income, from 92.5% of those from families with incomes less than 130% of FPL to 94.4% of those from families with incomes between 130 and 349% of FPL to 97.1% of those from families with incomes at or greater than 350% of FPL” (55). Therefore, programs like the ACA are needed to support families’ ability to access nutritionally dense foods or access supplements such as multi-vitamins.

Vitamin B12 is commonly found in animal products such as meat, fish, poultry, milk and eggs. Unlike potassium and folate, the larger percentage of vitamin B12 intake in this study (>90%) was from supplementation. Our review of available over-the-counter dietary supplements indicated that amounts of vitamin B12 contained in many products does often exceed the RDA. This may explain the findings that intake of vitamin B12 was over 2800% RDA before and after the ACA. Our study also found many Americans already consume the RDA of B12 from food alone (Table 2).

One of the main effects of the ACA was Medicaid expansion. In 2015, 29 states had expanded eligibility for their Medicaid programs, per the ACA initiatives, resulting in a 13% increase in Medicaid enrollment. Moreover, Medicaid and its affiliated program, the State Children’s Health Insurance Program (SCHIP), designed to provide insurance coverage for children whose families earn too much to qualify for Medicaid, expanded coverage by increasing the eligibility for pregnant and new mothers from 133% Federal Poverty Level to 138% (56). This resulted in decreases for the uninsurance rate for new mothers from 20.2 to 11.3% (2011, 2015), respectively, (57). This is consistent with the observed increase in the use of folate, mainly for pregnant people, and/or a multivitamin regimen as covered by Medicaid.

As food access issues in vulnerable communities continues to be a pervasive problem that is difficult to address, improving access to multivitamins or other nutrition supplements can be an alternative approach to ensuring appropriate nutrient intake for at-risk populations (58). However, shortages of primary care providers in these communities may impact the ability of individuals to receive nutrition counseling and gain access to prescription nutrient supplementation. In a 2017 report by the New England Journal of Medicine, Miller and Wherry reported that while Medicaid expansion increase coverage and access to care, post implementation, it was also associated with longer wait time for appointments (59). Further research is needed to determine the overall effectiveness of supplement use in this population to help achieve optimal nutrient intake, improve overall health, and reduce chronic disease, particularly in vulnerable populations where access to care and adequate nutrition remains an issue. This finding also highlights an opportunity for increasing access to nutrition intervention and counseling in community settings, especially in specific patient populations that stand to benefit even more from these interventions.

Our study has several limitations related to its cross-sectional design and the inherent biases associated with the use of patient self-reported data. Because the NHANES uses respondent panels that are updated every 2 years, we could not track the actual changes in individual behavior from prior to after the ACA. Instead, we used cross-sectional and longitudinal survey weights to extrapolate the results obtained from the two study cohorts to the US population. Furthermore, individual self-reported data on nutrient intake, primary care utilization and comorbid conditions could not be validated with direct tests and observations. Hence, we were limited in our ability to examine the role of access to healthcare services as a mediator of theassociation with ACA coverage expansion and the use of nutrition supplementation.

## 5 Conclusion

To our knowledge, this is the first study that examined the association of an expansion in a national health insurance on nutritional status of general population, with the special emphasis on the populations at greater risk for nutritional deficiencies – low-income individuals and patients with chronic conditions. Our findings suggest that the ACA expansion had a positive association with the population nutritional intake, mainly due to the increase in the use of dietary supplements but not food. Future studies should evaluate the long-term impact of the individuals ACA components (e.g., prevention programs, Medicaid expansion, prohibiting the denial of coverage to individuals with pre-existing conditions, among others) on the nutrient intake of the American population. More research is needed to inform accurate and culturally appropriate dissemination of public health messaging about the health benefits of adequate intake of key nutrients from food.

## Data availability statement

Publicly available datasets were analyzed in this study. This data can be found here: https://www.cdc.gov/nchs/nhanes/index.htm.

## Author contributions

AS, TT, KM, and KN: study conceptualization and design. HK, AS, JN, and KN: data curation and formal analysis. HK, AS, TT, and KN: writing – original draft. KN, KM, and SM: funding acquisition. HK, AS, KN, PD, KM, and SM: writing – review & editing. All authors approved the final version of the manuscript.
